# Application of Simodont virtual simulation system for preclinical teaching of access and coronal cavity preparation

**DOI:** 10.1371/journal.pone.0315732

**Published:** 2024-12-13

**Authors:** Yaru Wei, Zhengjun Peng

**Affiliations:** Guangdong Province Key Laboratory of Stomatology, Guanghua School of Stomatology, Hospital of Stomatology, Sun Yat-Sen University, Guangzhou, Guangdong, China; University of Puthisastra, CAMBODIA

## Abstract

To explore the quality, reliability, and practical effect of the Simodont virtual simulation training system in preclinical teaching of access and coronal cavity preparation for dental undergraduate students. Twenty dental undergraduate students from Guanghua School of Stomatology, Sun Yat-sen University, were recruited and randomly divided into two groups after theory training. The groups were allocated using the random number method and assessed for access and coronal cavity preparation skills using a standardized assessment form and the Simodont virtual simulation system operation manual. Baseline scores were recorded for each student. One group received training with the Simodont virtual simulation system, while the other used a conventional phantom simulator system. After training, skills were reassessed, and scores were recorded. The groups then switched training systems and were assessed again. All students completed a Teaching Questionnaire at the end of the training. Data was collected and analyzed. The mean score of students in the virtual simulation priority group (15.9 ± 0.56 points) and the phantom-simulator priority group (15.3 ± 0.40 points) was significantly higher than the baseline score (13.3 ± 0.63 points) and (13.1 ± 0.30 points) (*P* < 0.05), respectively. Furthermore, the mean score of students in the virtual simulation priority group after training with both systems (15.9 ± 0.56 points) was significantly higher than that of the students in the virtual simulation priority group alone (14.2 ± 0.62 points) (*P* < 0.05). The mean score was also significantly higher in the virtual simulation priority group of students (15.9 ± 0.56 points) trained with both systems than in the phantom-simulator priority group of students (15.3 ± 0.40 points) trained with both systems for the assessment (p < 0.05). The mean score of students in the phantom-simulator priority group (15.3 ± 0.40 points) after training with both systems was significantly higher than that of the students in the phantom-simulator priority group alone (14.3 ± 0.28 points) (*P* < 0.05). The questionnaire results showed that the students fully agreed that "the Simodont virtual simulation system has the characteristics of repeatability, multi-dimensionality, and multiple practices, and gives me more attention to details. However, they also noted that "it needs to be improved and upgraded to be closer to the conventional phantom-simulator system. Compared with the conventional phantom-simulator system alone, the preclinical teaching effectiveness of access and coronal cavity preparation could be significantly enhanced by incorporating the Simodont virtual simulation system alongside the phantom-simulator training system. The training sequence might influence this improvement.

## Introduction

Laboratory teaching is crucial for dental students to effectively acquire clinical skills to successfully transition into dentistry [1]. At present, the laboratory teaching of dental students in China still mainly relies on traditional teaching tools, such as the phantom-simulator training system. Students can acquire fundamental clinical skills through a phantom-simulator training system, including adjusting the patient’s position, utilizing traditional oral examination tools, selecting the pivot point during procedures, and tooth preparation. Both isolated and simulated teeth can be used in a phantom-simulator training system. However, the phantom-simulator training system still faces difficulties, such as the simple structure of the simulated teeth supporting the phantom-simulator model, poor operational feedback, high cost of use, and extended learning cycles. Compared to isolated teeth, the structural hierarchy of simulated teeth is not clearly defined. The tactile sensation during manipulation varies significantly. These challenges make it difficult to standardize dental education [1].

With the development of science and technology, the application of virtual simulation technology in medical laboratory education has been gradually recognized in recent years and has shown certain advantages [2]. Virtual simulation technology utilizes computer-generated 3D images to replicate the clinical environment, providing consistent training, enhancing teaching effectiveness, and enabling more objective evaluation [3–6]. In 2011, the "computerized virtual technology incorporating mechanical feedback" was introduced into the teaching of dentistry laboratories by developing the Simodont virtual simulation training system [7]. This system can offer precise digital simulation and mechanical control of oral treatment procedures, engaging tactile, visual, and auditory senses. It can assist students in mastering oral clinical skills such as caries removal, cavity preparation, cavity filling, and crown preparation accurately and realistically. The system provides benefits such as environmental protection such as medical contamination, repeatable practice, and high safety standards [8,9]. Its ultimate goal is to complement or, to a certain extent, replace existing physical training systems. In the last two years, with the continuous improvement of teaching modules and the redevelopment of new software, the digital virtual simulation training system has opened up a new field of teaching and training in endodontics, focusing mainly on access and coronal cavity preparation exercises. By setting different density parameters, this system can simulate the natural tactile sensation of enamel, dentin, and even carious cavities and root cementum, providing a clinical operation experience that cannot be achieved with isolated or simulated plastic teeth [10–12]. However, this system’s actual effects and application characteristics compared to the physical operating system still require further practical research.

Since the introduction of the Simodont virtual simulation system at the Guanghua School of Stomatology, Sun Yat-sen University in 2010, and its implementation in laboratory teaching, students have generally reported that the simulation effect of the system is realistic, and the operation is flexible and stable. In this study, the Simodont virtual simulation training system was utilized in the preclinical teaching of access and coronal cavity preparation for 2019 undergraduates at Guanghua School of Stomatology, Sun Yat-sen University. The effectiveness of the Simodont virtual simulation training system in preclinical teaching of access and coronal cavity preparation was assessed by comparing it with the conventional phantom-simulator training system. The aim was to explore the quality, reliability, and practical effect of the Simodont virtual simulation training system in preclinical teaching for dental undergraduate students.

## Materials and methods

### Simodont virtual simulation training system

**Fig 1:**
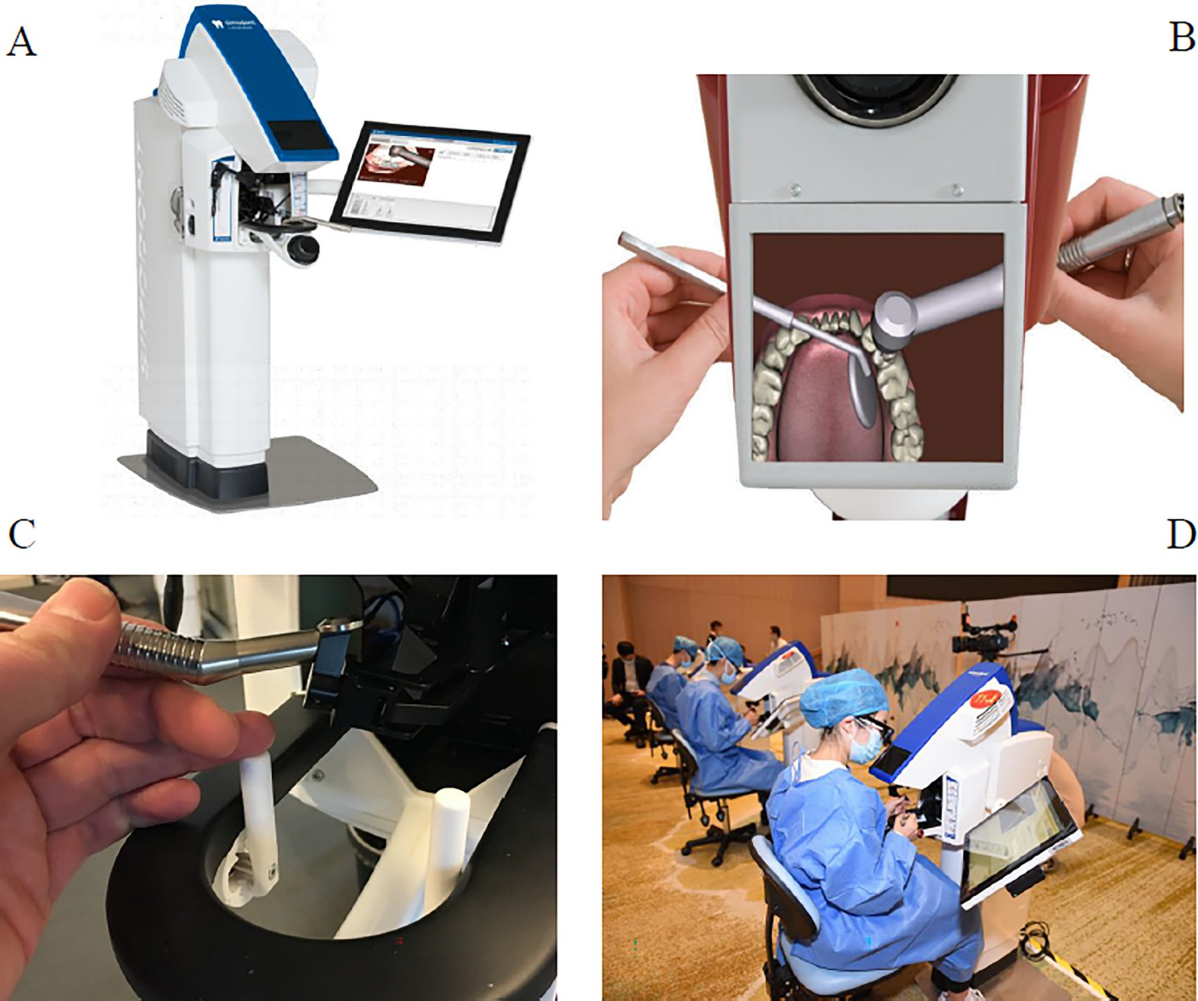
The Simodont virtual simulation system. The student operating system of Simodont virtual simulation system (A). 3D imaging of Simodont virtual simulation system (B). Operating platform of Simodont virtual simulation system (C). Students operate Simodont virtual simulation system (D).

The Simodont virtual simulation training system (**Fig 1**) was jointly developed by the Academic Centre for Dentistry at the Amsterdam Centre for Dentistry (ACTA, The Netherlands) and the Moog Company (USA). This system utilizes computer technology through digital simulation and a precise force-feedback device to create a virtual training system that closely resembles reality. It is designed for the preclinical training of dental students, allowing for intensive practice and assessment of operational skills. The system mainly consists of three-dimensional image generation and real-time tactile feedback (force feedback) of the integrated lifting host. Each component includes a touch screen, three-dimensional viewer, force feedback unit, foot pedal, simulation of drilling and grinding of dental tissues using the handpiece, operation of the bracket, three-dimensional mouse, simulation of the mouth mirror, three-dimensional glasses, simulation of the pivot bar, handpiece grip, and mouth mirror grip.

### Research object

**Fig 2:**
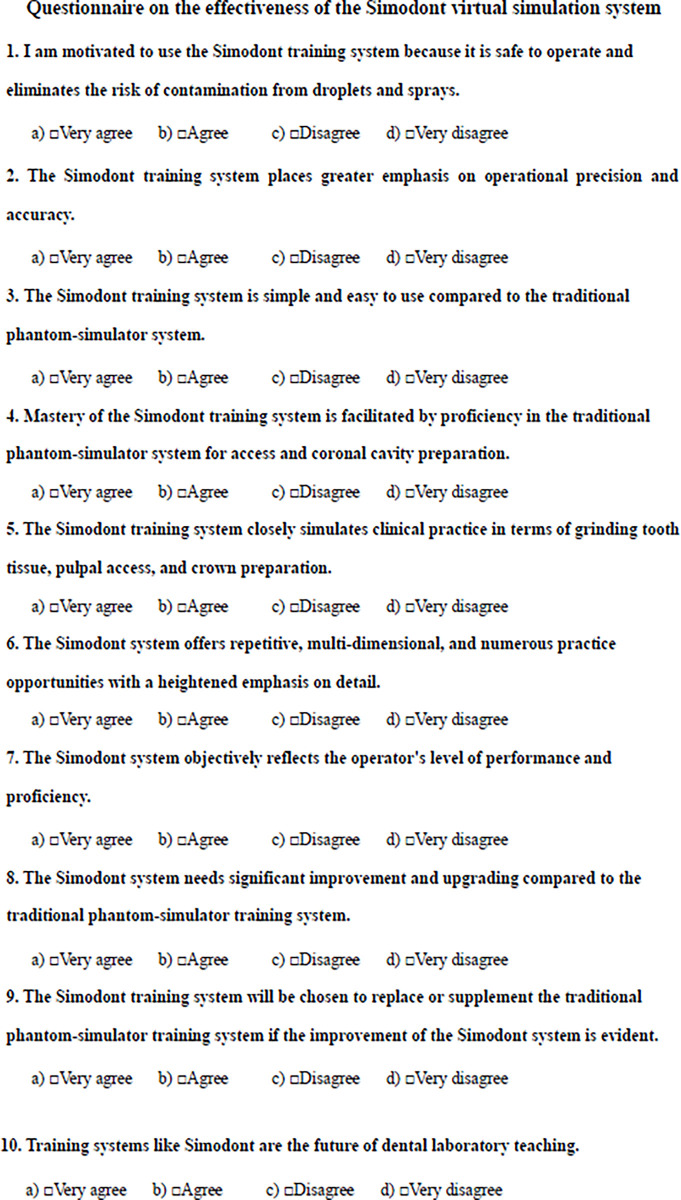
Flowchart of the experiment.

The undergraduate class of 2019, consisting of 20 students in total, at the Department of Stomatology, Guanghua School of Stomatology, Sun Yat-sen University, has commenced the preclinical training stage (**Fig 2**). All students have finished the relevant theoretical studies but have not engaged in clinical practice. The whole class volunteered to participate in this project study. The Hospital of Stomatology Ethics Committee approved the study, Sun Yat-sen University (No. KQEC-2024-89-01), which waived the need for review.

### Research methodology

1. Grouping: ten students with informed consent were randomly assigned to the virtual simulation priority group, and another ten students with informed consent were assigned to the phantom-simulator priority group using a random number table.
2. Pre-standardized training involves instructing students to review the relevant chapters of the theory course on access and coronal cavity preparation. They are also taught the critical points of operation and assessment criteria for access and coronal cavity preparation. The assessment criteria for access and coronal cavity preparation are designed to evaluate students’ performance and the outcomes of pulp opening. The former includes the correct choice of instruments, the way of holding them, and the proper movements and procedures; the latter includes whether the opening position, cavity shape, and the amount of tooth tissue are appropriate, whether the top of the pulp chamber is removed, whether the pulp chamber is appropriately shaped, whether the pulp chamber bottom is intact, and whether the root canal can be positioned. In addition, the Simodont Operator’s Manual is explained and illustrated to familiarize the student with the interface and procedures of the training system.
3. Training, Assessment, and Marking

Pre-training scoring (A): Before the start of the exercise, the two groups of students underwent a preliminary test on access and coronal cavity preparation skills. The operation involved simulating the preparation of the maxillary first molar on plastic teeth mounted on an imitation phantom simulator. The preparation of the phantom simulator consists of collecting isolated teeth, creating plaster casts, and preparing access and coronal cavities under rubber dam conditions using a phantom simulator. The examination lasted 18 minutes and was evaluated by three experienced endodontics teachers based on specific assessment criteria. The scores from this examination were recorded as pre-training scoring A. In the assessment scoring table (**Table 1**), seven examination points with a total score of 20 points were designed based on the clinical needs, considering the syllabus’s requirements. The data in this section were used to evaluate whether subgroups of students exhibited statistically significant differences regarding the rationale for access and coronal cavity preparation.

**Table 1:** Predetermined criteria of access and coronal cavity preparation.

Operation items and requirements	Score
Correct choice of instruments	0.5
Way of holding instruments	2.0
Correct movements and procedures	2.5
Opening position, cavity shape, and the amount of tooth tissue	4.0
Removal of the top of pulp chamber	4.0
Shape and integrity of pulp chamber bottom	4.0
Positioning of the root canal	3.0
Totals	20.0

Post-training scoring (B/C): The virtual simulation priority group utilized the Simodont system for access and coronal cavity preparation, while the phantom-simulator priority group practiced on isolated teeth using the conventional phantom-simulator training system. Both groups had a training duration of 2 hours. Following the training, participants completed a simulated case of access and coronal cavity preparation on a plastic tooth model of the maxillary first molar within 18 minutes using a phantom simulator. This work was assessed by three experienced endodontics teachers based on predetermined criteria, resulting in the stage 1 training score (B/C). Teachers provided prompt feedback to students using PowerPoint presentations and the phantom simulator and encouraged self-study and self-correction. Post-training scoring (B/C) data were used to assess the impact of a single operating system on access and coronal cavity preparation in both groups of students.

Interactive training and scoring (D/E): Two groups of students swapped training systems and practiced for 2 hours. After the training session, each student completed a case involving access and coronal cavity preparation of a maxillary first molar on a simulated plastic tooth within 18 minutes using a phantom simulator. Three experienced endodontics teachers assessed the performance according to predetermined criteria. The results contributed to evaluating the stage 2 training (D/E). Teachers provided prompt feedback on the assessments to the students using PowerPoint presentations and the phantom simulator, guiding them in self-study and self-correction. Interchangeable operating systems were assessed to determine their impact on access and coronal cavity preparation and whether the sequence of operations influenced the results.

4 Questionnaire survey

**Fig 3:**
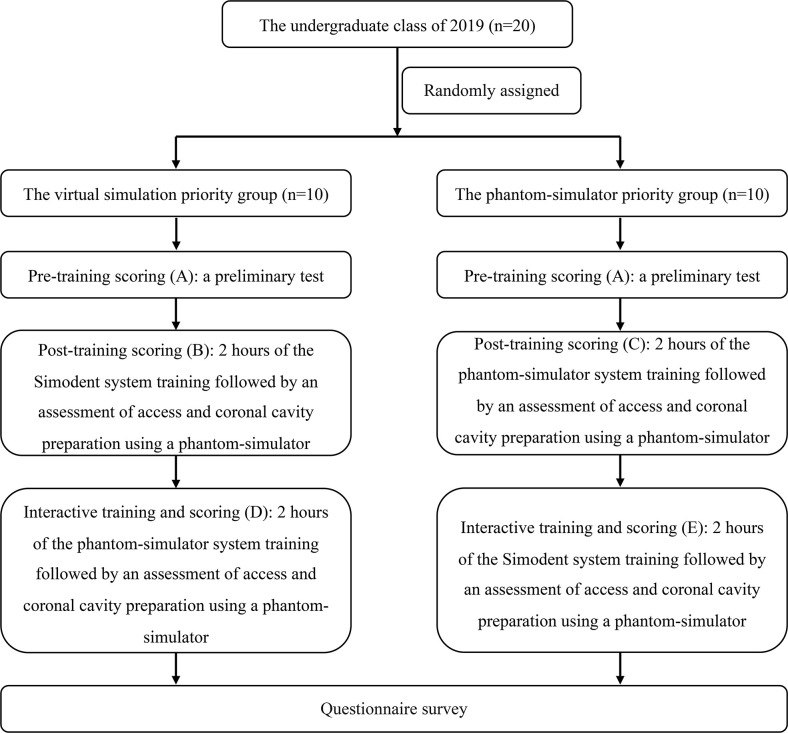
Questionnaire on the effectiveness of the Simodont virtual simulation system.

The questionnaire, designed for the 2019 academic year, assesses the effectiveness of the Simodont system in teaching access and coronal cavity preparation, as depicted in Fig 3. Additionally, it was evaluated for both content and structural validity. This questionnaire is crafted to gauge students’ experiences with the Simodont system, identify its strengths and weaknesses, and lay the groundwork for enhancing the system to enhance its support for preclinical teaching. The main points include the willingness to use the Simodont system, the advantages and shortcomings of the conventional phantom-simulator training system, and the need for improvement. The students are required to complete the questionnaire within the specified time.

### Data analysis

The data were entered into IBM SPSS Statistics 26.0 software, and the appraisal scores for access and coronal cavity preparation were analyzed for normality using the Shapiro-Wilk test. The data were found to follow a normal distribution. A two-independent-sample t-test was used to compare access and coronal cavity preparation assessment scores in the Simodont and the phantom-simulator priority groups. One-way ANOVA with the LSD/Dunnett T3 method was used to compare the appraisal scores of different training modalities within the same group pairwise. The significance level of the test was set at α = 0.05 (two-sided).

## Results

### Comparison of the teaching effectiveness of access and coronal cavity preparation

The statistics of single assessment scores from **Table 2** found that the Simodont priority group scored significantly higher than the phantom-simulator priority group in pre-training scores for correct movements and procedures, opening position, cavity shape, the amount of tooth tissue, and positioning of the root canal (*P* < 0.05). The phantom-simulator priority group scored higher than the Simodont priority group in removing the top of the pulp chamber and in shaping and integrity of the chamber bottom (*P* < 0.05). The single-system training and assessment scores found that the Simodont priority group scored higher than the phantom-simulator priority group in positioning the root canal (*P* < 0.05). The phantom-simulator priority group scored higher than the Simodont priority group in opening position, cavity shape, the amount of tooth tissue, and removal of the top of the pulp chamber (*P* < 0.05). In the assessment scores following the training of the two systems, it was observed that the Simodont priority group achieved higher scores than the phantom-simulator priority group (*P* < 0.05) incorrect movements and procedures, shaping and integrity of pulp chamber bottom, and positioning of the root canal. The phantom-simulator priority group scored higher than the Simodont priority group in the opening position, cavity shape, and amount of tooth tissue (*P* < 0.05).

**Table 2:** Scoring of access and coronal cavity preparation training assessment for undergraduate stomatology students ($\bar{x}$± s).

Group	Assessment grouping	Number of persons per group	Correct choice of instruments (0.5 points)	Way of holding instruments (2 points)	Correct movements and procedures (2.5 points)	Opening position, cavity shape, and the amount of tooth tissue (4 points)	Removal of the top of pulp chamber (4 points)	Shape and integrity of pulp chamber bottom (4 points)	Positioning of the root canal (3 points)	totals (20 points)
Group of the Simodont priority	A	10	0.5	2	1.71 ± 0.16	2.22 ± 0.15	2.12 ± 0.15	2.12 ± 0.13	2.62 ± 0.11	13.3 ± 0.63 d
	B	10	0.5	2	1.90 ± 0.15	2.38 ± 0.16	2.31 ± 0.14	2.60 ± 0.18	2.50 ± 0.13	14.2 ± 0.62 e
	D	10	0.5	2	2.03 ± 0.11	2.46 ± 0.09	2.84 ± 0.14	3.07 ± 0.17	2.95 ± 0.14	15.9 ± 0.56 f
Group of the phantom-simulator priority	A	10	0.5	2	1.54 ± 0.11	2.01 ± 0.11	2.40 ± 0.08	2.38 ± 0.08	2.28 ± 0.08	13.1 ± 0.30 g
	C	10	0.5	2	1.95 ± 0.11	2.63 ± 0.12	2.62 ± 0.08	2.49 ± 0.12	2.12 ± 0.08	14.3 ± 0.28 h
	E	10	0.5	2	1.92 ± 0.10	2.79 ± 0.12	2.77 ± 0.09	2.67 ± 0.08	2.69 ± 0.10	15.3 ± 0.40 i
t value a			/	/	2.64	3.49	5.06	5.36	7.78	0.99
P value a			/	/	<0.05	<0.05	<0.05	<0.05	<0.05	0.33
t value b			/	/	0.86	3.86	5.94	1.59	7.78	0.51
P value b			/	/	0.41	<0.05	<0.05	0.13	<0.05	0.61
t value c			/	/	2.24	6.78	1.29	6.89	4.89	2.33
P value c			/	/	<0.05	<0.05	0.21	<0.05	<0.05	<0.05

Note: A is a pre-training assessment. B is a post-training assessment using only the Simodont system. C is a post-training assessment using only the phantom-simulator system. D involves using the Simodont system initially, followed by the phantom-simulator system after training and assessment. E involves using the phantom-simulator system first, followed by the Simodont system after training and assessment. ^a^ denotes the comparison of the Simodont priority group A scores with phantom-simulator priority group A scores. ^b^ denotes the comparison of the Simodont priority group B scores with phantom-simulator priority group C scores. ^c^ denotes the comparison of the Simodont priority group D scores with phantom-simulator priority group E scores. In the context of this study, ^d, e, f, g, h, and i^ represent comparisons between various assessment subgroups within the same group. When the same letters are used, it indicates that there are no statistically significant differences between those subgroups. Conversely, if different letters are used, it signifies statistically significant differences (P < 0.05). Additionally, "/" denotes full scores, and t-tests were not conducted.

The statistical analysis of the total scores found that all the student’s assessment scores for access and coronal cavity preparation were significantly higher after training than before training (*P* < 0.05). The total score of students in the Simodont priority group after training with both systems for access and coronal cavity preparation (15.9 ± 0.56 points) was significantly higher than that of the students in the Simodont priority group alone (14.2 ± 0.62 points) (*P* < 0.05). The total score for access and coronal cavity preparation was also significantly higher in the Simodont priority group of students (15.9 ± 0.56 points) trained with both systems than in the phantom-simulator priority group of students (15.3 ± 0.40 points) trained with both systems for the assessment (*P* < 0.05). The total score of students in the phantom-simulator priority group (15.3 ± 0.40 points) after training with both systems for access and coronal cavity preparation was significantly higher than that of the students in the phantom-simulator priority group alone (14.3 ± 0.28 points) (*P* < 0.05).

The results above indicate that when using a single training system, there is no significant difference between the training outcomes of the conventional phantom-simulator system and the new Simodont system for access and coronal cavity preparation. Moreover, combining both training systems is more effective than using a single system. However, the sequence in which the training systems are combined can impact the assessment scores. The Simodont priority group achieved a higher overall experimental score than the phantom-simulator priority group.

### Student evaluation of the Simodont system

Regarding the willingness to use the Simodont system for access and coronal cavity preparation exercises, 90% (18/20) of the students agreed that the system was safe to operate and free from contamination, such as droplets and sprays. Additionally, 60% (12/20) agreed that the Simodont system placed more emphasis on precision and accuracy and expressed their willingness to use the system. Regarding experience, only 10% (2/20) of the students agreed that the Simodont system was more accessible to operate or use than the conventional phantom-simulator system. However, 70% (14/20) of the students decided that it was easier to master the Simodont system for access and coronal cavity preparation after mastering the preparation of the cavity with the conventional phantom-simulator system. Only 10% (2/20) of the students agreed that the Simodont system was closer to clinical practice in grinding tooth tissue, pulpal access, and crown preparation.

90% (18/20) of the students agreed that the Simodont system provides repetitive, multi-dimensional, and multiple practice opportunities with a greater focus on detail. 30% (6/20) of the students decided that the Simodont system objectively reflects the operator’s level of performance and proficiency. 80% (16/20) of the students agreed that the Simodont system needs significant improvement and upgrading compared to the conventional phantom-simulator training system. If the Simodont system is significantly improved, 60% (12/20) of the students agreed that the Simodont system could replace or supplement the conventional phantom-simulator training system for access and coronal cavity preparation. 70% (14/20) of the students decided that a simulation system, like the Simodont system, is the future trend for teaching in dental laboratories. Overall, innovative approaches are more likely to capture students’ attention. Regarding usability, the new system requires additional hardware and software upgrades to enhance its integration with traditional experiments.

## Discussion

Preparing access and coronal cavities is the first step in root canal treatment. The standardization and precision of this procedure directly influence the efficiency of the subsequent treatment and can even affect the overall treatment outcome [13]. Therefore, emphasizing this concept to students and conducting sequential exercises in laboratory teaching is the first step in assisting students in developing a precise understanding of access and coronal cavity preparation. Preclinical training for access and coronal cavity preparation is based on a combined phantom-simulator training system using isolated and simulated plastic teeth. The effectiveness of laboratory teachings on access and coronal cavity in isolated teeth is influenced by various factors. The availability of isolated teeth for collection is limited, and the unique morphology of the pulpal cavity, along with its anatomical structure, adds complexity for beginners to understand and perform the procedures [14]. Moreover, these factors can lead to biased assessment results, diminishing teaching and evaluation reliability. Simulating plastic teeth is costly due to significant differences in anatomical structure and material compared to natural teeth. It is challenging to replicate the authentic tactile sensation of enamel and dentin while preparing access and coronal cavities. During phantom-simulator training for access and coronal cavity preparation, it is challenging for the teacher to observe the students’ practice process and operational details in real-time due to the limited operation field. Consequently, pinpointing specific issues and effectively resolving them becomes problematic. Moreover, the cutting operation of dental tissues during the phantom-simulator training is irreversible and unrepeatable. This makes it difficult to replicate the practice to achieve the desired teaching effect of proficiency in operational skills.

The virtual simulation training system is based on virtual reality technology [15], which aims to simulate routine and complex cases in actual clinics. This allows students to practice repeatedly without incurring additional costs. The teaching models can be reproduced and reversed using 3D virtual simulation interactive software and a virtual classroom. In addition, the virtual simulation training system can observe and record the operational process’s three-dimensional motion and force data in real-time, allowing for playback, observation, and analysis [16]. This standardizes and quantifies the teaching, training, and assessment processes, facilitating the scientific evaluation of teaching quality. In the field of dentistry, various virtual simulation training systems have been introduced, covering endodontics [6], periodontics [17], prosthodontics [5], oral implantology [18], maxillofacial surgery [3], and other specialties. At present, the use of virtual simulation technology for virtual laboratory teaching has become a trend in the development of dental education, aiming to replace or supplement the deficiencies in physical teaching partially.

In recent years, research on the Simodont system in endodontics teaching has primarily focused on caries teaching, including cavity preparation training and scoring system evaluation [19–22]. The Simodont system itself comes with advanced teaching functions, including automatic scoring of caries preparation, crown preparation, complex case analysis, and network teaching. Further design and exploration are needed to utilize these advanced functions fully for dental laboratory teaching. This study found that combining the Simodont and phantom-simulator training systems was more effective than using a single training system alone. Additionally, the teaching effect was influenced by the sequence in which the training systems were used.

The combined use of the two training systems increased students’ practice time and proficiency in the practical operations of access and coronal cavity preparation. Furthermore, students’ curiosity and seriousness about the novelty of the virtual training system further contributed to the effectiveness of the teaching and learning. This study showed that training with the Simodont system, followed by the phantom-simulator system, significantly improved the assessment scores for access and coronal cavity preparation compared to using the Simodont system alone. The improvements were observed in terms of the removal of the top of the pulp chamber. This may be because the plastic teeth used in the assessment closely resemble the isolated teeth utilized in the Simodont system training regarding pulpal location, morphology, and texture. Therefore, supplementing the Simodont system with isolated teeth manipulation practice before engaging in virtual practice facilitates students in understanding the position of the pulpal opening, cavity shape, amount of tooth tissue, and debridement of the top of the pulpal chamber. Training with the Simodont system followed by the phantom-simulator system resulted in higher assessment scores regarding ideal pulp chamber shape, integrity of the pulp chamber floor, and positioning of the root canal opening compared to using the phantom-simulator system alone. This effect may be attributed to the compelling force feedback the Simodont system provides. It gives students a clear sensation of descent while preparing the crown of the medullary chamber, enhancing their perceptual awareness. This, in turn, leads to improved application and comprehension of the relevant procedures during subsequent practice with the phantom-simulator system. In addition, the Simodont system can magnify minor errors in operational practice, prompting students to pay closer attention to operational details. Combined with multi-dimensional, repetitive practice and reinforced practice on the Simodont system, this improves access and coronal cavity preparation outcomes.

In this study, the results of the teaching questionnaire revealed that students agreed with the Simodont system being "reproducible, multidimensional, offering multiple practice opportunities, and providing more attention to detail." However, students unanimously agreed that "The Simodont system should be enhanced to resemble a phantom-simulator training system more closely." As we all know, the Simodont system can provide virtual patient information, simulate real-life diagnostic and treatment processes, develop students’ diagnostic and treatment ideas, and provide a safe and clean practice environment.

However, there are some shortcomings of the Simodont system, such as the high initial cost of installation, the need for professional training before using the Simodont system, the inability to simulate effective patient-doctor communication, the difficulty of imitating real-life isolated teeth, the slow speed of expanding and upgrading the software functions, and the discomfort caused by wearing 3D glasses for an extended period. The tactile sensations of enamel, dentin, and bone can only be accurately simulated by precisely setting and calibrating the system’s density parameters [4]. If the cases in the Simodont system can be continuously improved, the instruments are constantly updated, and then jointly applied with the conventional phantom-simulator training system to complement each other’s strengths and weaknesses, it can truly achieve the goal of partially replacing or supplementing physical teaching. This combined approach is expected to enhance teaching effectiveness significantly.

It is necessary to acknowledge this work’s limitations. According to the statistical sample size calculation, it barely meets the experimental requirements. However, the sample size is too small, affecting the experiment’s scientific validity. Therefore, subsequent experiments should increase the sample size to verify the experimental conclusions. The assessment criteria provide objective ratings; the following experimental design should minimize the influence of subjective factors on the stability of the experimental results. This study mainly performed learning and testing. Retention tests can measure memory retention and learning effects, which assess participants’ long-term mastery of knowledge [23]. Therefore, retention tests should be further implemented along with larger sample sizes.

## Conclusion

Compared with the conventional phantom-simulator training system, the Simodont system, when used in conjunction with the phantom-simulator training system, can effectively enhance the teaching effectiveness of access and coronal cavity preparation. The effectiveness is influenced by the sequence of their joint application, offering new ideas and methods to improve the quality of teaching in the initial stages of access and coronal cavity preparation. At the same time, the virtual simulation system is a method based on physical sensation, somewhat detached from physical awareness and perception, and can serve as an auxiliary teaching tool.

## Supporting information

S1 File Ethical review comments, raw data and analysis results. (RAR)
